# Translation, cross-cultural adaptation into Brazilian Portuguese and electronic migration of the WERF EPHect endometriosis patient questionnaire

**DOI:** 10.61622/rbgo/2025rbgo40

**Published:** 2025-09-08

**Authors:** Gabriele Barbosa Anelli, Rafael Zucco de Oliveira, Juliana Meola, Daniela Michelazzo, Ricardo Perussi-e-Silva, Julio Cesar Rosa-e-Silva, Christian Becker, Nilufer Rahmioglu, Lone Hummelshoj, Stacey Missmer, Krina Zondervan, Omero Benedicto Poli-Neto

**Affiliations:** 1 Universidade de São Paulo Hospital das Clínicas of the Ribeirão Preto Medical School Ribeirão Preto SP Brazil Hospital das Clínicas of the Ribeirão Preto Medical School, Universidade de São Paulo, Ribeirão Preto, SP, Brazil.; 2 Universidade de São Paulo Ribeirão Preto Medical School Department of Obstetrics and Gynecology Ribeirão Preto SP Brazil Department of Obstetrics and Gynecology, Ribeirão Preto Medical School, Universidade de São Paulo, Ribeirão Preto, SP, Brazil.; 3 Universidade de São Paulo Laboratory for Translational Data Science Ribeirão Preto SP Brazil Laboratory for Translational Data Science, Universidade de São Paulo, Ribeirão Preto, SP, Brazil.; 4 University of Oxford Nuffield Dept of Women's and Reproductive Health Endometriosis CaRe Centre Oxford United Kingdom Endometriosis CaRe Centre Oxford, Nuffield Dept of Women's and Reproductive Health, University of Oxford, United Kingdom.; 5 University of Oxford Wellcome Center for Human Genetics United Kingdom Wellcome Center for Human Genetics, University of Oxford, United Kingdom.; 6 World Endometriosis Research Foundation London United Kingdom World Endometriosis Research Foundation, London, United Kingdom.; 7 University of Michigan Department of Obstetrics and Gynecology Ann Arbor MI United States Department of Obstetrics and Gynecology, University of Michigan, Ann Arbor, MI, United States.

**Keywords:** EPHect, Endometriosis, Pelvic pain, Cross cultural comparison, Electronic health records, Data collection, Surveys and questionnaires

## Abstract

**Objectives:**

To translate and cross-culturally adapt the standard version of the WERF EPHect EPQ into Brazilian Portuguese and migrate it to an electronic version using REDCap.

**Methods:**

The study included 120 women aged 18–50 years, with half completing the paper version and half the electronic version. The sample comprised 80 individuals with chronic pelvic pain (CPP), including 60 with endometriosis, recruited from a tertiary referral centre, and 40 women from a primary healthcare unit with no diagnosis of CPP or endometriosis. Illiterate or cognitively impaired individuals were excluded. The electronic migration was implemented in REDCap.

**Results:**

The Brazilian Portuguese EPQ-S demonstrated linguistic accuracy and cultural equivalence to the original English version. The paper version consisted of 35 pages, and 86.7% of respondents commented on its length. Despite this, it was well-received, although minor difficulties were observed among participants with lower education levels. The electronic version provided a more accessible experience, with a significantly shorter completion time (52.1 ± 13.2 minutes) compared to the paper version (70.9 ± 21.4 minutes). Both formats exhibited similar rates of missing data for questions related to symptoms and contraceptive use.

**Conclusion:**

This study successfully translated, adapted, and migrated the WERF EPHect EPQ-S into Brazilian Portuguese using REDCap. The findings underscore the linguistic and cultural challenges of such adaptations and highlight the electronic version's advantages in accessibility, efficiency, and participant satisfaction. This research supports the development of cross-cultural assessment tools for endometriosis research and healthcare delivery.

## Introduction

Endometriosis is characterised by the presence of functional endometrial tissue, including glands and stroma, outside the uterine cavity. It is a common condition worldwide, potentially affecting around 10% of women and those assigned female at birth (hereafter referred to as women) of reproductive age.^(1)^ Although identified in at least 4% of asymptomatic women undergoing tubal sterilisation, it is frequently associated with painful symptoms such as dysmenorrhea, dyspareunia, dyschezia, acyclic pelvic pain, and infertility.^(2)^ These outcomes have negatively impacted various aspects of individuals’ lives,^(3)^ thereby imposing a significant personal burden as well as economic and healthcare challenges.^(4)^ The heterogeneity of the condition encompasses not only clinical symptoms but also various lesion subtypes (superficial peritoneal, ovarian cysts, deep endometriosis),^(5)^ diverse pathogenetic mechanisms,^(6)^ transcriptomic factors,^(7)^ and genomic influences,^(8)^ among others. This complexity poses significant challenges in both diagnosis and treatment approaches for the condition.

There is consensus among major international societies that a presumptive diagnosis of the disease warrants initiating clinical treatment due to the absence of pathognomonic signs or symptoms. Hormonal therapy is generally advocated as the first-line approach for managing pain, given its comparable effectiveness to surgical interventions but with lower complication rates and costs.^(9)^ However, these medications prove effective in only about two-thirds of patients^(10)^ and demonstrate limited long-term efficacy,^(11,12)^ occasionally causing undesirable side effects. This treatment approach does not extend to infertility treatment, as it lacks beneficial effects in this regard.^(13)^ Consequently, a significant number of women undergo surgery for disease management, which can remove visible endometriotic lesions but does not cure the disease.^(14)^ Despite initial clinical improvements post-surgery, recurrence is common, particularly without postoperative hormonal therapy.^(15)^

Endometriosis Phenome and Biobanking Harmonization Project (EPHect) with the aim "to enable large-scale, cross-centre, epidemiologically robust research into the causes of endometriosis, novel diagnostic methods, and better treatments through the development of an international consensus on: standardised detailed clinical and personal phenotyping (phenome) data to be collected from women with endometriosis and controls, to improve patient and disease characterization; and standard operating procedures (SOPs) for banking of biological samples from women with endometriosis and controls, with respect to collection, transport, processing, and long-term storage".^(16–19)^ One of the developed tools was the EPHect Clinical Questionnaire (EPQ), which guides the recording of clinical characteristics of participants, especially those relevant to endometriosis diagnosis, and more. It is available in two versions, a complete (Standard - S) version and a short-form (Minimum - M) version. The difference between the two versions is that the first includes additional questions focusing on detailed information about age strata and the inclusion of questions related to body shape, also stratified by age groups. For this reason, we chose to use the complete version in this study. Any future adaptation for the use of the reduced version would be easily implemented without the need for additional studies. Currently, 65 institutions in 24 countries are registered as user centres for the available tools (https://endometriosisfoundation.org/centres-using-ephect-tools/). In Brazil, only one institution is a participant so far, the Ribeirão Preto Medical School of the University of São Paulo. Considering that Brazil is the largest country in Latin America and the seventh most populous country in the world, with over 200 million inhabitants, we consider the translation and cross-cultural validation of the EPQ-S instrument into Brazilian Portuguese to be fundamental, along with promoting its use, at least in research settings. Certainly, this would be of great value within the global context of efforts for endometriosis research.

Parallel to this, numerous studies have demonstrated that the adoption of electronic data capture (EDC) systems enhances the efficiency, accuracy, and cost-effectiveness of data registration, storage, and management in research.^(20)^ Furthermore, the use of reliable patient-reported outcome (PRO) data entered directly through electronic means has been increasingly prevalent. This shift from paper to electronic formats has been propelled not only by the widespread availability of internet access but also by reduced operational costs, validation efforts,^(21,22)^ and improved compliance.^(23)^ Electronic versions have demonstrated a variety of advantages over paper formats.^(24)^ Our institution has adopted REDCap (Research Electronic Data Capture), a secure web-based software platform utilising PHP + JavaScript programming languages and MySQL database engine designed to facilitate data capture for research studies.^(25,26)^ REDCap provides: 1) an intuitive interface for validated data capture; 2) audit trails to monitor data manipulation and export processes; 3) automated procedures for seamless data downloads into common statistical packages; and 4) protocols for data integration and interoperability with external sources,^(27)^ among other features.

The aim of this study was to translate and cross-culturally adapt the standard version of the WERF EPHect EPQ into Brazilian Portuguese and to migrate it to an electronic version using REDCap.

## Methods

Initially, we communicated our study aims to the coordinating group of the WERF EPHect initiative via email. The process of translation and cross-cultural adaptation of the WERF EPHect EPQ-S to Brazilian Portuguese was based on the propositions of Beaton et al.^(28)^ For the migration from the paper version (p-EPQ) to the electronic version (e-EPQ), we also communicated with WERF, and the first author of the study^(19)^ provided us with the original English REDCap template. For implementation, we closely followed the recommendations of the International Society for Pharmacoeconomics and Outcomes Research (ISPOR).^(29)^

**Figure 1:**
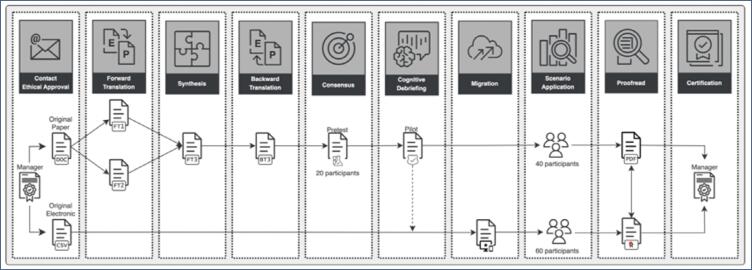
Summary of the steps of translation, cross-cultural adaptation into Brazilian Portuguese, and electronic migration of the WERF EPHect Endometriosis Participant Questionnaire. This figure schematically illustrates the flow of the processes carried out and detailed in the Methods section

Notes: ‘CSV’ = Original custom template of the electronic version of the instrument in REDCap provided by the manager in .csv format; ‘DOC’ = Original paper version provided by the manager in .docx format; ‘FT1’ = Forward-translated version produced by a medical gynaecologist specialized in the field; ‘FT2’ = Forward-translated version produced by a bilingual professional with a postgraduate degree in linguistic studies; ‘FT3’ = Forward-translated consensus version derived from the two previous versions; ‘BT3’ = Back-translation for a professional who is a native English speaker with Brazilian Portuguese as her second language; ‘PDF’ = Final paper version formatted and shared with the manager in .pdf format; ‘R’ = Custom template of the Brazilian Portuguese electronic version of the instrument implemented in REDCap.

The instruments Pain Catastrophizing Scale (PCS) and the short form of the McGill Pain Questionnaire (SF-MPQ), both included in the WERF EPHect EPQ-S, had previously been translated and validated into Brazilian Portuguese.^(30,31)^ Therefore, we did not retranslate them, instead using the available validated Brazilian Portuguese versions. Figure 1 summarily outlines all the steps taken.

We included a total of 120 women aged between 18 and 50 years in the study. Of these, 80 had chronic pelvic pain (60 with surgically confirmed endometriosis) and were recruited prospectively from the HCFMRP-USP Pelvic Pain Centre, an interdisciplinary tertiary referral service for women with chronic pelvic pain. The remaining 40 participants were apparently healthy women, also prospectively recruited from the Social and Community Medical Center of Vila Lobato, a primary healthcare unit. Illiterate women or those with cognitive problems that prevented reading or minimal understanding of written language were not eligible. Participants were instructed to complete the paper document using block letters. The electronic version was filled out on a desktop or laptop.

The instrument was independently translated by two translators, both of whom are native Brazilian Portuguese speakers fluent in English. A forward-translated version (FT1) was produced by a medical gynaecologist specialised in the field, while a second version (FT2) was produced by a bilingual professional with a postgraduate degree in linguistic studies and extensive expertise in translation but without a medical or clinical background. Both translators were familiar with the specific cultural nuances and terminologies of the target population.

In this phase, the two versions were comparatively analysed by the translators and a bilingual panel consisting of three clinical gynaecologists with expertise in endometriosis/pelvic pain and primary care. They assessed the clarity, accuracy, and cultural relevance of the translations and provided feedback. Thus, a consensus version (FT3) was reached, which was used in the next phase.

Version FT3 was translated back into English (BT3) by a third independent translator, who was unaware of the original version. This professional is a native English speaker with Brazilian Portuguese as her second language. She has lived in Brazil for over 40 years, has no clinical or medical background, and had no prior knowledge of the original instrument.

An expert committee was constituted, comprising eight clinical professionals and the two translators without a clinical background. The clinical professionals included three expert gynaecologists in pain and endometriosis (GBA, JCRS, OBPN), a gynaecologist working as a primary care provider (DM), two biologist researchers in endometriosis (JM, RZO), and a clinical database administrator and interviewer for clinical studies (RSP). They performed the comparison and harmonisation between the original version, forward translation, and backward translation to identify and resolve any discrepancies or cultural biases. The expert panel played a crucial role in ensuring conceptual and linguistic equivalence. They produced a pretest version of the questionnaire.

The pretesting phase involved administering the instrument to a small sample of the target population to assess its comprehensibility, clarity, and cultural appropriateness. This version of the instrument incorporated modifications and/or adaptations recommended by the expert panel review, except for ethnic questions (questions F2, F3, F4 presented in supplement 1). It was administered to 20 women: 10 had chronic pelvic pain with imaging/surgically confirmed endometriosis and were attended to in the specialised service; the remaining 10 were apparently healthy women attending routine gynaecological consultations in the primary care service. Women with different educational levels and age groups were included in both groups. Participants read the instructions and questions and answered the paper questionnaire in the presence of an interviewer. Questions and difficulties were recorded. At this stage, the time for completion was not timed. Feedback from the participants was collected to further refine the instrument and produce the pilot version.

In this phase, we considered pretesting performance and proposed modifying a few questions (questions F2, F3, F4 presented in supplement 1) of the instrument to ensure its relevance and suitability for the target culture. We took into account equivalent expressions widely used by the Brazilian Institute of Geography and Statistics (IBGE), an official government agency responsible for providing statistical data and information about the country. This involved modifying or adding items and adapting response options. The adaptations were made in consultation with experts from the target culture to preserve the instrument's properties. All doubts and discrepancies of interpretation considered of minor relevance were discussed in a new round by the expert committee. We also discussed some adaptations in the document format, without any additional changes in content. After review and rephrasing to ensure equivalence with the original instrument, the final version of the p-EPQ was produced.

As mentioned earlier, the original REDCap template was obtained in CSV format. We proceeded with the replacement of the instrument labels, keeping the original variable names. In our view, this facilitates communicability in data extraction and integration processes among participating centres. Implementation version (REDCap 9.8.2-© 2020 Vanderbilt University), current version (REDCap 13.5.1-© 2023 Vanderbilt University). Modifications and/or adaptations from the original instrument were reported.

The final versions of the p-EPQ and the e-EPQ were administered to an additional 40 and 60 participants, respectively. In this phase, the questionnaire completion time was timed. Questions raised by participants and users were recorded.

This was a critical step in preparing final documents as it ensures error correction, enhances clarity and readability, projects professionalism, boosts credibility and trustworthiness, reinforces the proposal's image, avoids legal or academic consequences, and ultimately saves time and costs. By investing time in thorough proofreading, authors can present polished and accurate documents that effectively convey their intended message to the readers.

The Brazilian Portuguese version of the WERF EPHect EPQ-S in both paper and electronic formats was made available to the project managers.

We used the Mann-Whitney test (Wilcoxon rank-sum test) to compare the questionnaire completion time between groups.

Subsequently, the study received approval from the Ethics Committee of the Hospital das Clínicas of the Ribeirão Preto Medical School (HCFMRP-USP) at the University of São Paulo 3.088.003 (*Certificado de Apresentação de Apreciação Ética:* 04016818.0.0000.5440).

## Results

The participants’ characteristics are presented in more detail in table 1.

**Table 1:** Characterization of the participants in the study of validation, cross-cultural adaptation and electronic migration of the WERF EPHect EPQ-S to the Brazilian Portuguese version

Characteristics		EPQ paper version (n=60)			EPQ electronic version (n=60)		
		CPP with endometriosis n(%) (n=30)	CPP without endometriosis n(%) (n=10)	Apparently healthy n(%) (n=20)	CPP with endometriosis n(%) (n=30)	CPP without endometriosis n(%) (n=10)	Apparently healthy n(%) (n=20)
Age in years, median (interquartile range)		35(31.2-38)	39(36.2-42.8)	35(30-40.5)	36(32-39.8)	39.5(38.2-42)	35.5(29.8-42.2)
	18-29 years	3(10.0)	1(10.0)	4(20.0)	4(13.3)	0(0.0)	5(25.0)
	30-39 years	24(80.0)	5(50.0)	10(50.0)	18(60.0)	5(50.0)	8(40.0)
	40-49 years	3(10.0)	4(40.0)	6(30.0)	8(26.7)	5(50.0)	7(35.0)
BMI in kg/m^2^, median (interquartile range)		27.6(22.9-30.7)	25.8(25.1-26.8)	25.2(24.4-29.9)	26.7(23.8-31.2)	26.2(22.7-34.5)	24.4(23.4-26.3)
Ethnic group							
	white	11(36.7)	3(30.0)	10(50.0)	15(50.0)	2(20.0)	8(40.0)
	mixed	16(53.3)	5(50.0)	6(30.0)	9(30.0)	5(50.0)	8(40.0)
	black	3(10.0)	2(20.0)	4(20.0)	6(20.0)	3(30.0)	4(20.0)
Education (> 12 years)		13(43.3)	5(50.0)	5(25.0)	9(30.0)	2(20.0)	3(15.0)
Menstruation (last 3 months)							
	Irregularity	7(23.3)	2(20.0)	-(-)	6(20.0)	2(20.0)	-(-)
	Moderate/heavy menstrual bleeding	7(23.3)	2(20.0)	-(-)	8(26.7)	2(20.0)	-(-)
	No complain	16(53.3)	6(60.0)	20(100.0)	16(53.3)	6(60.0)	20(100.0)
Hormonal contraceptive							
	Progestogen-only pill	15(50.0)	6(60.0)	7(35.0)	17(56.7)	7(70.0)	5(25.0)
	Combined pill	3(10.0)	2(20.0)	7(35.0)	6(20.0)	3(30.0)	7(35.0)
	Other	8(26.7)	1(10.0)	1(5.0)	7(23.3)	0(0.0)	2(10.0)
	None	4(13.3)	1(10.0)	5(25.0)	0(0.0)	0(0.0)	6(30.0)
Nulliparous		15(50.0)	2(20.0)	7(23.3)	12(40.0)	3(30.0)	6(30.0)
Infertility		15(50.0)	1(10.0)	-(-)	13(43.3)	1(10.0)	-(-)
Pain during menstruation		25(83.3)	6(60.0)	-(-)	23(76.7)	2(20.0)	-(-)
Pain during vaginal intercourse		22(73.3)	9(90.0)	-(-)	24(80.0)	6(60.0)	-(-)
Previous abdominal surgery		30(100.0)	4(40.0)	3(15.0)	30(100.0)	3(30.0)	-(-)

BMI = body mass index; CPP = chronic pelvic pain; Ethnic group = black and afro descendant considered together; Menstrual bleeding = on average

The analysis of Brazilian literature, social networks, and the terminology used by various communication outlets and professional organisations such as the Brazilian Federation of Gynecology and Obstetrics Associations (Febrasgo) and the Brazilian Society of Endometriosis, as well as the expert panel review, suggested that all concepts within the Brazilian Portuguese EPHect EPQ-S had the same meaning as the original English version. It adequately addressed aspects related to personal information and lifestyle, reproductive health, symptoms, comorbidities, previous surgeries, and treatments in our target population. The backward translation phase produced a very similar translation. The final version exhibited excellent semantic equivalence (grammatical and vocabulary) and was culturally adapted.

The paper version, formatted in portable document format (PDF), comprises 35 pages. The modifications and adaptations incorporated through the expert panel review are presented in Supplement 1. As anticipated, questions F2, F3, and F4 regarding ethnicity, while semantically understandable, were critical and needed cultural adaptation after pretesting. The majority of participants (85.0% [17/20]) reported that it was difficult to answer them accurately. In addition to these modifications, we started each section on a new page and attempted to adapt all tables to fit on a single page. However, this was not possible for questions B1, C15, C28, C41, D4, E1, and E2. The electronic version is hosted at https://redcap.fmrp.usp.br.

### Cognitive testing on the paper version

We observed that the majority of participants found the document long (86.7% [52/60]). However, almost all of them (98.3% [59/60]) expressed satisfaction with the translation and linguistic accuracy of the adapted Brazilian Portuguese questionnaire. In general, the questions were found to be clear, understandable, and in line with the original intent, including for participants with lower educational levels, regardless of age. A small percentage of participants (23.3% [14/60]), mainly those with lower educational levels, needed to return to previous pages to recall some questions, especially in multi-page tables.

During the pretesting phase, 65.0% of participants (13/20) answered questions they should have skipped or showed some anxiety about skipping questions, especially when it was necessary to move to more than one page. In the pilot version, after the incorporation of explicit instructions, there was an improvement in these rates, but they were still considered high (47.5% [19/40]). Questions assessing details about symptoms or characteristics during the participants’ life course (A3, C15, C28, C41) had a high proportion of missing information, lack of recall, or reports of inaccuracy in completion (78.3% [47/60]). Interviewers had the impression that these questions might have been answered inaccurately.

Twenty-six point seven percent (16/60) of participants had doubts about the name of the contraceptive used or its composition (Question A4), and 43.3% (26/60) stated that they didn't remember all the hormones they had used, particularly when more than 10 years had passed since use. In 6.7% of cases (4/60), it was not possible to decipher the written name of the hormone used. Only 2 participants (3.3% [2/60]) chose not to answer the questions related to sexual relations due to feeling a certain degree of discomfort or embarrassment. Concerning completion time, we noted that the paper version required an average completion time of 70.9 ± 21.4 minutes. Participants with the highest educational level (higher/postgraduate education) completed the questionnaire more quickly (61.0 ± 20.3 minutes) compared to others (77.0 ± 20.0 minutes) (p = .013).

### Cognitive testing in the electronic version

The electronic version contains a participant identification section, study information, and the interview. We set the ID, participant name, survey date, and date of birth as identifiers. This is in accordance with the General Data Protection Law in force in the country. We used REDCap's native translation tool for Brazilian Portuguese, enabling the use of yes/no fields and other information in language accessible to our population. All exclusively descriptive fields containing only images were deleted. Images were inserted into fields with respective guidance texts after enabling REDCap's native "rich format editor" function.

Only 6.7% of participants reported spontaneously that the questionnaire was long (4/60). There were no limitations regarding the use of computer resources. All participants were skilled in using computers. There were no improper completions of questions that needed to be skipped due to automatically imposed variable conditions. Questions requesting details about symptoms or characteristics during the participants’ life course (A3, C15, C28, C41) still had a high proportion of lack of recall, or reports of inaccuracy in completion (65.0% [39/60]). Regarding question A4, 31.7% [19/60] of participants had doubts about the name of the contraceptive used or its composition, with a disagreement between the name and the type of hormone recorded in 8.3% of cases (5/60). Additionally, 35.0% of them (21/60) stated that they didn't remember all the hormones they had used, particularly when more than 10 years had passed since use. These data are similar to those observed in the paper version.

**Figure 2:**
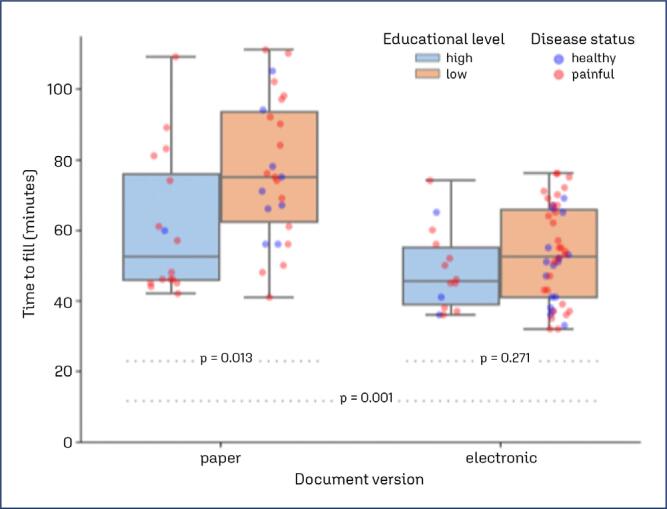
Comparison of questionnaire completion time by health condition and educational level: paper vs. electronic versions. This boxplot illustrates the time taken to complete the questionnaire, comparing paper and electronic versions. The data is grouped by health condition and participants’ educational level. The plot highlights the variation in completion times across different groups and formats, providing insights into how educational background and health status influence response times

Unlike the paper version, there were no difficulties in reading the written information, although we identified minor spelling errors in the description of some contraceptive trade names. The average completion time was 52.1±13.2 minutes, significantly shorter than the time spent on the paper version (p < .001). Participants with the highest educational level (full elementary and high school) completed the questionnaire more quickly (48.6±11.7 minutes) compared to others (53.1±13.6 minutes), but this difference was not significant (p = .271). Figure 2 shows the questionnaire completion time highlighting the educational level and health condition of all participants. Supplement 2 describes the modifications and adaptations incorporated into the Brazilian Portuguese electronic version of the WERF EPHect EPQ-S in the REDCap template.

The version of the questionnaire developed in this study, as well as in other languages, is available free of charge upon request. We have also attached the Brazilian Portuguese version as supplementary material (Supplement 3). The original World Endometriosis Research Foundation Endometriosis Phenome and Biobanking Harmonisation Project Endometriosis Participant Questionnaire (WERF EPHect EPQ), both the Standard and Minimum versions, as well as other tools, can be accessed directly via the following link: https://www.ephect.org/tools/patient-questionnaire.

## Discussion

Our study detailed the stages involved in translating and culturally adapting the WERF EPHect EPQ-S, along with its migration to the REDCap platform, emphasising key considerations and recommendations, as well as challenges encountered. The study found that the concepts in the Brazilian Portuguese version aligned with the original English version, covering various aspects pertinent to the target population, such as personal information, lifestyle, reproductive health, symptoms, comorbidities, surgeries, and treatments. This equivalence was confirmed during the backward translation phase.

The paper version was presented in a 35-page PDF format. Specifically, questions related to ethnicity required cultural adaptation after pretesting due to challenges from participants in providing accurate responses. Despite its length, the paper version received high satisfaction regarding translation quality and clarity among participants, with minor difficulties noted among those with lower educational levels. Notably, participants experienced anxiety when skipping questions spanning multiple pages.

In contrast, the electronic version provided a more accessible experience. Utilising the REDCap platform, it enabled native translation of instructions and user-friendly formats. Unlike the paper version, the electronic format reduced completion time and presented no difficulties for interviewers in reading information, regardless of participants’ educational level. However, regardless of the version used, questions about symptoms and life course characteristics continued to show instances of potential inaccuracies in reports. Similarly, occasional uncertainties persisted regarding contraceptive names and compositions, with discrepancies in recorded hormone types in some cases.

This study revealed an average completion time ranging from 48 to 77 minutes, which aligns with the Danish^(32)^ and Turkish^(33)^ versions but exceeds that of the Canadian French version.^(34)^ While we lack a comprehensive explanation for this, our impression is that women spent significant time on questions requiring detailed or specific past memories. This may induce cognitive overload, characterised by mental fatigue and reduced concentration. Lengthy or complex questionnaires pose a risk of patients providing incorrect or inaccurate responses.^(35)^ However, despite its length, the questionnaire provides valuable information, such as assessing catastrophizing, which can be beneficial for therapeutic planning.^(36)^

Participants with higher educational levels completed the electronic questionnaire slightly faster than those with lower levels, although the difference was not significant. Based on these findings, we recommend prioritising the electronic version whenever feasible, with caution regarding the interpretation of some provided information due to potential imprecision. Furthermore, the electronic version minimises typing errors, reduces issues with data transfer from paper to electronic databases, allows for redundant storage, enhances information recovery capabilities, eliminates physical damage concerns, facilitates easier database auditing, and ensures uniform and swift data provision. Moreover, it may incorporate automated controls to mitigate incomplete responses or issue alerts for missing data, as demonstrated in this template. This observation is significant as it allows participants to revisit and provide any missing information, although careful consideration should be given to enforcing electronic completion to avoid respondents providing inaccurate information when uncertain.

Furthermore, we propose discussing potential additions for future revisions of the questionnaire, particularly focusing on enhancing its scope and relevance. One crucial aspect to consider is the incorporation of objective instruments for measuring psychological symptoms. Research has shown that symptoms like anxiety and depression significantly impact the lives of women with endometriosis and chronic pelvic pain, potentially influencing the effectiveness of therapeutic interventions.^(37)^ Various Patient-Reported Outcome (PRO) measures are available for this population, as highlighted in a recent review.^(38)^

Another significant consideration is the role of central sensitization (CS) in the pathophysiology of pelvic pain associated with endometriosis, especially regarding central nervous system involvement.^(39)^ While clinical instruments for measuring CS have faced scrutiny, its association with poorer pain outcomes post-endometriosis surgery has been documented.^(40)^

We achieved strong linguistic accuracy and addressed most concerns regarding potential ambiguities and confusing terminology. Ensuring conceptual equivalence in translation studies is complex and demanding, requiring not only linguistic precision but also capturing underlying meanings, nuances, and cultural contexts from the source to the target language. Complete equivalence can be challenging due to language and cultural disparities. Brazil's cultural diversity, influenced by its historical, ethnic, racial, and social factors in a continental-sized country, necessitates careful consideration in adapting instruments. While we aim for comprehensive cultural representation, localised adaptations may become necessary over time.

In today's globalised context, the demand for valid and reliable instruments across diverse cultural and linguistic settings is crucial. Translating and culturally adapting instruments for cross-cultural research is pivotal to ensuring measurement integrity and equivalence. While the WERF EPHect EPQ-S is available in multiple languages (https://endometriosisfoundation.org/ephect/#ephect-sops-questionnaires-forms), disclosing the translation and adaptation processes, as exemplified in our study and others,^(32–34)^ is vital for maintaining accuracy, cultural sensitivity, patient safety, and ethical standards in healthcare delivery and research. It fosters improved communication, collaboration, and equity in healthcare provision.

In addition to acknowledging the practical challenges of administering the complete instrument in busy outpatient settings, where factors such as limited consultation time, physical space constraints, internet access difficulties, long queues, and lower educational levels may hinder routine use, we wish to emphasise that, although the EPHect initiative was primarily designed to support large-scale, internationally collaborative and epidemiologically robust research in endometriosis, it also offers significant promise for clinical practice. Standardising the collection of clinical and phenotypic data not only enhances our understanding of this complex condition but also bridges the gap between clinical care and collaborative research, ultimately improving patient outcomes. It is also important to note that some limitations may be addressed or minimised by adopting the short-form version. Nevertheless, we have shared this perspective with other authors, who also recognise the potential of EPHect in both research and clinical environments.^(41)^

## Conclusion

In conclusion, our study successfully adapted the Brazilian Portuguese version of the EPHect EPQ-S questionnaire, with electronic formatting significantly enhancing completion time and accessibility. Despite ongoing challenges, our research offers valuable insights into the linguistic and cultural adaptation of such instruments for specific populations. These findings contribute to advancing knowledge in questionnaire translation and adaptation, guiding healthcare professionals and researchers in developing effective cross-cultural assessment tools.

## Supplement 1 Modifications and adaptations incorporated into the Brazilian Portuguese version of the WERF EPHect EPQ-S

**Supplement 1:** Modifications and adaptations incorporated into the Brazilian Portuguese version of the WERF EPHect EPQ-S

Question	Comments/original question	Modification/adapted version of the question
A2	1. Trade name is not recommended for national agencies; 2. HRT abbreviation; 3. Lack of examples available in the Brazilian market and unified health system; 4. Participants tended to continue answering the questions A2.2 and A2.3 in the sequence presented; 5. DD, MM, YYYY abbreviations.	1. We deleted trade names; 2. We wrote in full hormone replacement therapy; 3. We added patch and ring options for this and other questions (A2.4); 4. We have added additional information "Please, skip to question A2.4"; 5. We have substituted abbreviations by the words "DAY", "MONTH" and "YEAR".
A4	1. Trade name is not recommended for national agencies; 2. Lack of combined injectables available in the Brazilian market and unified health system; 3. Danazol is only available in Brazil for oral use (although as a rule it is not used as a contraceptive method).	1. We deleted trade names; 2. We added combined injectable option; 3. We deleted the guidance "please specify if used vaginally or orally" following the option "Danazol".
A6	1. In Brazil, we formally use the term "acne", but it is popularly known as "pimples".	1. We included the popular term "pimples" in parentheses to represent what most participants understood.
C4	1. Lack of diclofenac as example, a drug available and commonly used in the Brazilian market and unified health system;	1. We added diclofenac as an option of pain-killer; 2. We changed the order of examples for: "e.g. aspirin, diclofenac, paracetamol/acetaminophen, ibuprofen, naproxen".
C8	1. Usually, the Brazilian scales present the information "No pain" and "Worst imaginable pain" below the numerical scale itself.	1. We changed the information "No pain" and "Worst imaginable pain" for the bottom of the scale.
C10	The same as in question C8.	The same as in question C8.
C13	The same as in question C8.	The same as in question C8.
C14	The same as in question C4.	The same as in question C4.
C15	The same as in question C8.	The same as in question C8.
C20	The same as in question C8.	The same as in question C8.
C21	The same as in question C8.	The same as in question C8.
C27	The same as in question C8.	The same as in question C8.
C28	The same as in question C8.	The same as in question C8.
C32	The same as in question C4.	The same as in question C4.
C33	The same as in question C8.	The same as in question C8.
C39	The same as in question C8.	The same as in question C8.
C40	The same as in question C4.	The same as in question C4.
C41	The same as in question C8.	The same as in question C8.
D8	1. Participants tended to continue answering the questions D8.1 to D8.5 in the sequence presented.	1. We have added additional information "If not, please, skip to question D9".
D8.3	1. The original was "D8.3 How old were you when you first had symptoms? _____ years old or tick here □ if you never had symptoms"	1. We rephrased the question for: "D8.3 Did you have any symptoms? ___No ___Yes If Yes, D.8.3.1 How old were you when you first had symptoms?"
E1	1. The order and groups of drugs in the "Pain relief drug table" were a bit confusing for our participants. Most of them did not know how to distinguish between types of non-steroidal anti-inflammatory drugs.	1. We have incorporated ibuprofen into the "other anti-inflammatory analgesics" category; 2. We modified the order of the rows according to the most used "Type of drug" in the country.
F1	1. DD, MM, YYYY abbreviations.	1. We have substituted abbreviations by the words "DAY", "MONTH" and "YEAR".
F2	F2. How would you describe your ethnic origin? □ American Indian or Alaskan native □ Asian/Pacific/Oriental □ Black □ Black African □ African American □ Black Caribbean □ Native Hawaiian or other Pacific Islanders □ White □ North/West European □ East European □ South European □ North American □ Other: ___________(please specify) □ Mixed Race □ Other: ___________(please specify)	F2. Were you born in Brazil? □ No □ Yes If No: F2.1. Since when do you live in the country? F2.2. In which country were you born? F2.3. Are you a refugee or undocumented immigrant? □ No □ Yes
F3	F3. Do you consider yourself to be Spanish/Hispanic/Latina? No □ Yes	F3. Among the following alternatives, do you recognize or identify yourself as being of any colour or race? □ afrodescendant □ indigenous □ yellow □ black □ white □ brown □ other: ___________(please specify)
F4	F4. What is your major ancestry? (Please check one). □ Scandinavian □ African □ Irish / Celtic / British □ Asian □ Other Northern European □ Southern European / Mediterranean □ Eastern European □ Other (Please specify): ___________ □ South American □ Don't know □ Central American or Caribbean	F4. Indicate your family origin from the following alternatives: □ African □ Central American □ European □ Indigenous □ Jewish □ North American □ Eastern European, Syrian, Lebanese, Armenian □ Southeast Asian □ South American (except Brasil) □ Other ___________(please specify) □ Don't know
F6	F6. What is the highest level of education you have attained (with certificate)? □ Primary/grade school □ Lower secondary/middle school □ Upper secondary/high school □ Post-secondary not university/some college or vocational school □ University □ Postgraduate	F6. What is the highest level of education you have attained (with certificate)? □ Primary school/primary education □ Secondary school/elementary school □ High school □ Non-university technical training □ University □ Postgraduate
F7	1. "feet" and "inches" units	1. We kept only the option with the international notation system (centimetres).
F8	1. "pounds" and "stones" units	1. We kept only the option with the international notation system (kilograms).
F9	The same as in question F8.	The same as in question F8.
F10	The same as in question F8.	The same as in question F8.
F15	F15. During the last 12 months, what was your average time per week spent on each of the following recreational activities? □ Walking or hiking outdoors (include walking to work) □ Jogging (slower than 10 minutes/mile) □ Running (10 minutes/mile or faster) □ Bicycling (include stationary machine) □ Calisthenics/aerobics/aerobic dance/rowing machine □ Tennis, squash, racquetball □ Lap swimming □ Other aerobic recreation (e.g., lawn mowing)	F15. During the last 12 months, what was your average time per week spent on each of the following recreational activities? □ Walking or hiking outdoors (include walking to work) □ Jogging (slower than 10 kilometres/hour) □ Running (10 kilometres/hour or faster) □ Bicycling (include stationary machine) □ Calisthenics/aerobics/aerobic dance/rowing machine □ Tennis, squash, racquetball, soccer, volleyball □ Lap swimming □ Other aerobic recreation (e.g., lawn mowing)
F17	F17. Do you drink any alcohol? □No □ Yes If yes: F17.1. During an average week, how much do you drink of each of the following? (Please note exact numbers, not ranges such as 1-3) Type of alcohol (serving size) Beer/lager/cider (half pints (284 ml) Sherry/vermouth/port (50 ml) Wine (175 ml) Spirits (25 ml) Other (Please specify)	F17. Do you drink any alcohol? □No □ Yes If yes: F17.1. During an average week, how much do you drink of each of the following? (Please note exact numbers, not ranges such as 1-3) Type of alcohol (serving size) Beer/chopp/cider (300 ml) Sherry/vermouth/port/liquor (50 ml) Wine (a glass of 150-175 ml) Distillates/cachaça/vodka/whiskey (25 ml) Other (Please specify)

## Supplement 2 Modifications and adaptations incorporated into the Brazilian Portuguese electronic version of the WERF EPHect EPQ-S in the REDCap template

**Supplement 2:** Modifications and adaptations incorporated into the Brazilian Portuguese electronic version of the WERF EPHect EPQ-S in the REDCap template

Question	Modification/ adaptation/ significative differences between paper and electronic versions
A3	The table is initially hidden. We keep the formatting as in the source electronic file. The question starts with a question about natural menstruation. If the selection is "Yes", the other questions referring to the selected age group are displayed below.
A4	The table is initially hidden. We keep the formatting as in the source electronic file. The question starts with a question about a previous pregnancy. If so, drill down options are displayed progressively.
B4	The table is initially hidden. We keep the formatting as in the source electronic file. The question starts with a question about seeking fertility treatment. If so, drill down options are displayed progressively.
C15	The table is initially hidden. We keep the formatting as in the source electronic file. The question begins with a question about pelvic pain during menstruation by age group. Detailing options are displayed progressively, if the response confirms the existence of pain. Questions referring to pain intensity by age group were presented as lines in a matrix with a single answer format.
C23	Questions C23.1, C23.2, C23.3 and C23.4 in the paper version offered the options "Yes" and "No" for the questions "Was intercourse/vaginal penetration attempted during this time frame?" and "If yes, was it more painful at this time than other times?". In the electronic version, they had their layout adapted to a matrix with a single response format for three columns with the following statements: 1) "You tried to have vaginal intercourse/penetration in this period of time and it was more painful than at other times", 2) "You attempted vaginal intercourse/penetration in this time period and it was not more painful than at other times", and 3) "Not applicable, vaginal intercourse/penetration was not attempted in this period".
C28	The table is initially hidden. We keep the formatting as in the source electronic file. The question begins with a question about pelvic pain during or in the 24 hours after intercourse/vaginal penetration by age group. Detailing options are displayed progressively, if the response confirms the existence of pain.
C41	The table is initially hidden. We keep the formatting as in the source electronic file. The question starts with a question about the frequency of pelvic pain by age group. Breakdown options are displayed progressively, if the response confirms a frequency considered relevant.
C43	Each item was represented as an individual question in the source electronic file. We chose to change the layout, keeping exactly the same content. We chose to represent them in a matrix with the row index representing "other pains" and the columns presenting the 3 original response options "No", "Yes, in last month", and "Yes, more than 1 month ago" with single response permission only.
D2	The second block of the table is initially hidden and the questions begin with a question about the existence of the indicated medical issue. If the answer is affirmative, a field is displayed for entering the age in the diagnosis.
D4	The second block of the table (indicating the age at diagnosis) is initially hidden and the questions begin with a question about the indicated surgical procedure. If the answer is affirmative, a field is displayed for entering the number of surgeries, when appropriate, and the age and reason for the procedure.
D10	In the original electronic file, there were individual variables for each condition presented. We chose to include a question "Before answering the questions below, tell us if you have a sister" followed by a group of matrix for each condition of interest (endometriosis and chronic pelvic pain) whose row index represents the relatives and the options represented "No", "Yes", "Do not know", single answer only allowed. We added a condition to not display the "sister" option in the matrix if the answer to the previous question was "No".
E1	The table is initially hidden. We keep the formatting as in the source electronic file. The question begins with a question about the use of a particular drug and, in case of an affirmative answer, the detailing options are progressively displayed.
E2	The same as in question E1.
F2	The same as in the paper version.
F3	The same as in the paper version.
F4	The same as in the paper version.
F6	The same as in the paper version.
F7	The same as in the paper version.
F8	The same as in the paper version plus the inclusion of guidance for recording weight in kilograms with one decimal place.
F9	The same as in the paper version plus the inclusion of guidance for recording weight in kilograms with one decimal place.
F10	The same as in the paper version plus the inclusion of guidance for recording weight in kilograms with one decimal place.

All questions where multiple answers were allowed, we added the following information: "Please, check all that apply".
